# Suicidal ideation and attempts among people with severe mental disorder, Addis Ababa, Ethiopia, comparative cross-sectional study

**DOI:** 10.1186/s12991-018-0193-3

**Published:** 2018-06-01

**Authors:** Bereket Duko, Getinet Ayano

**Affiliations:** 1 Faculty of Health Sciences, College of Medicine and Health Sciences, Hawassa University, P. O. Box 1560 Hawassa, Ethiopia; 2 Research and Training Directorate, Amanuel Mental Specialized Hospital, Addis Ababa, Ethiopia

**Keywords:** Suicidal ideation, Suicidal attempt, Schizophrenia, Bipolar disorder

## Abstract

**Background:**

People with severe mental disorders are associated with increased risk of suicide and suicide attempts compared to the general population. In low and middle-income countries, research concerning suicide attempts and completed suicide among people living with severe mental disorder is limited. The objective of this study was to assess suicide and attempts in people with severe mental disorder at Amanuel Mental Specialized Hospital, Addis Ababa, Ethiopia.

**Methods:**

Institution-based cross-sectional study was conducted in August–September 2016. Patients with schizophrenia and bipolar disorder were selected using systematic random-sampling technique. The composite international diagnostic interview was used to assess suicide that was administered by psychiatry professionals. Substance use disorder was assessed through face-to-face interviews using structured clinical interview of DSM-IV.

**Results:**

A total of 542 (272 schizophrenia + 270 bipolar disorder) patients were included in the study. One hundred nineteen (43.75%) of schizophrenic participants and 128 (47.1%) of bipolar participants have suicidal ideation. Fifty-six (20.7%) of schizophrenic participants and 58 (21.3%) of bipolar participants have suicidal attempt. Among the schizophrenic and bipolar patients who had suicidal ideation, 31.8 and 32.60% had co-morbid substance use disorder, respectively.

**Conclusion:**

In this study, which was performed in Ethiopia, suicidal ideation and attempt were shown to be common problems in people with schizophrenia and bipolar disorder. Co-morbid substance use disorder was a more frequent phenomenon among patients with suicidal ideation and attempt. Attention should be given to screen and assess suicidal ideation and attempt in persons with schizophrenia and bipolar disorder.

## Background

Suicide is a huge but largely preventable health problem causing almost half of all violent deaths and resulting in one million fatalities each year, as well as economic costs in billions of dollars. Estimates suggest that suicide could rise to 1.5 million by 2020. Globally, suicide represents 1.4% of the global burden of diseases [1]. Suicide is usually a cause of great distress to victim, family, friends, and community and largely to the nation [2, 3].

According to different studies among all suicides over 90% of are explained by mental disorders [4–9] mostly mood disorders, alcohol and substance use disorders [9–12].

A recent review of the literatures estimated that up to 50% of schizophrenic patients attempt suicide and up to 13% of all deaths due to suicide are attributable to schizophrenia [13]. Compared to the general population (suicide prevalence about 1%), people with schizophrenia have a more than eightfold increased risk of suicide [14]. Suicide is the major cause of premature death among individuals with schizophrenia. Evidences indicated that up to 10% of patients with schizophrenia die by suicide [15–17]. Being young, male, and in the early years of the illness and having a history of multiple previous episodes or previous suicide attempts are the common risk factors for suicide in schizophrenia [18–21]. A substantial percentage of patients with schizophrenia also attempt suicide, with estimates of lifetime occurrence ranging from 18 to 55% [8].

Evidences indicated that persons with bipolar disorder are 30 times more likely to make a suicide attempt during their lifetime compared to those with no psychiatric disorder [22]. Close to one-third of persons with bipolar disorder attempt suicide [23, 24]. Researchers estimate that in the general population 29% of bipolar patients made at least one suicide attempt during their lives. In clinical samples, 25–56% of the patients with BD report at least one suicide attempt during their lives and 10–19% die by suicide [22–24]. A number of factors have been reported to be associated with the occurrence of suicide attempts in bipolar disorder and co-morbid substance use disorders (SUDs) [23, 25–27] is among those factors.

In persons with severe mental disorders co-morbid substance use disorders (SUD) are very common throughout the course of illness, with an estimated prevalence of 50–60% [28–31]. Nicotine and alcohol use disorders are particularly common among persons with severe mental disorders [8, 9]. Substance use disorder co-morbidity is eventually associated with worse outcome and higher suicidal risk [29, 30].

Evidences have shown that people with severe mental disorders (SMD) are at higher risk of suicide. However, in low- and middle-income countries (LMIC), including Ethiopia there is limited research concerning suicide attempts and suicide ideations in people with severe mental disorders (SMDs). The objective of this study was to assess suicide and suicide attempts in people with schizophrenia and bipolar disorder.

## Methods

### Study setting and population

Institution-based cross-sectional study was conducted in August 2016 at Amanuel Mental Specialized Hospital, Addis Ababa, Ethiopia. Amanuel Mental specialized hospital is the only hospital in Ethiopia giving services for mental health for long time. A total of 542 patients; 272 patients with the diagnosis of schizophrenia and 270 with bipolar disorder were included in the study. Study participants were included using systematic random-sampling technique.

### Inclusion and exclusion criteria

All patients with established DSM-IV diagnoses of schizophrenia and bipolar disorder who had treatment follow-up assessment were included in this study. Suicidal gesture or attempt was defined as a self-inflicted act associated with intent to die or use of a method with potential for lethality.

### Data collection instruments

Demographic variables were collected using semi-structured questionnaire. Data were collected by trained psychiatry professionals. The composite international diagnostic interview (CIDI) was administered by psychiatry professionals and used to assess suicide. Substance use disorder was assessed through face-to-face interviews using structured clinical interview of DSM-IV (SCID).

### Data processing and analyses

The statistical program for social science (SPSS version 20) was used for data analyses. Socio-demographic (age, sex, marital status, areas of residence, religion, education) and clinical factors (diagnosis, history of alcohol, cannabis, nicotine and khat abuse or dependence) was analyzed and reported using words, tables and charts.

## Results

### Socio-economic and demographic characteristics

A total of 572 patients; 270 patients with the diagnosis of schizophrenia and 272 with bipolar disorder were included in the study. The mean age of the respondents was 32.62 (± SD = 9.43) and 33.71 (± SD = 9.35) years for bipolar and schizophrenic participants, respectively. Among the total participants (bipolar and schizophrenia), 107 (39.3%) and 105 (38.8%) of participants had completed secondary educational level, respectively. Regarding income, the average monthly family income was 1450 (± SD = 648.50) and 1463 (± SD = 647.93) Ethiopian birr with respect to bipolar and schizophrenic participants (Table 1).

**Table 1:** Sociodemographic characteristics of people with severe mental disorders (schizophrenia, *n* = 270 and bipolar disorder, *n* = 272) Amanuel Hospital, Addis Ababa, Ethiopia, August, 2016

Characteristics	Schizophrenia		Bipolar disorder	
	Frequency	%	Frequency	%
Sex				
Male	186	68.8	196	72.1
Female	84	31.1	76	28.9
Age in years				
20–27	105	38.8	101	37.2
28–38	96	35.6	98	36
39–53	69	25.6	73	26.8
Marital status				
Single	157	58.2	159	58.5
Married	73	27	79	29
Separated	14	5.2	14	5.1
Divorce	26	9.6	20	7.4
Place of residence				
Urban	186	68.9	182	66.9
Rural	84	31.1	90	33.1
Religion				
Orthodox	168	62.3	166	61
Muslim	73	27	81	29.8
Protestant	24	8.8	19	7
Catholic	5	1.9	6	2.2
Educational level				
No school	30	11.1	27	9.9
Primary	78	28.9	86	31.6
Secondary	105	38.9	105	38.6
Higher education	57	21.1	54	19.9
Occupation				
Government employee	23	8.5	28	10.2
Private employee	89	33	87	32
Merchant	56	20.6	57	21
Unemployed	23	8.5	22	8.1
Student	59	21.9	53	19.5
Others	20	7.5	25	9.2
Monthly income				
300–1000	106	39.2	107	39.3
1001–1900	92	34.2	91	33.5
1901–3000	72	26.6	74	27.2
Ethnicity				
Amhara	63	23.3	58	21.3
Tigray	40	14.8	38	14
Oromo	102	37.8	100	36.8
Gurage	55	20.4	64	23.5
Others	10	3.7	12	4.4

### Suicidal ideation and attempt in patients with severe mental disorders

One hundred nineteen (43.75%) of schizophrenic participants and 128 (47.1%) of bipolar patients had suicidal ideation. In addition to this, 56 (20.7%) of schizophrenic participants and 58 (21.3%) of bipolar participants have suicidal attempt, respectively (Table 2).

**Table 2:** Distribution of patients with severe mental disorders by suicidal ideation and attempt (schizophrenia, *n* = 270 and bipolar disorder, *n* = 272) Amanuel Mental Specialized Hospital, Addis Ababa, Ethiopia, August, 2016

Suicidal behavior	Schizophrenia		Bipolar disorder	
	Yes	No	Yes	No
Suicidal ideation	119 (43.75%)	151 (56.25%)	128 (47.1%)	144 (52.9%)
Suicidal attempt	56 (20.7%)	214 (79.3%)	58 (21.3%)	214 (78.7%)

### Substance use disorders in patients with schizophrenia and bipolar disorders

Regarding khat, 137 (50.3%) of bipolar and 125 (36.6%) of schizophrenic patients had used in their life time. Concerning alcohol, 107 (39.1%) of bipolar and 99 (36.6%) schizophrenic patients had used in their life time. From schizophrenic patients, 130 (48.1%) and bipolar patients 86 (31.6%) had poly substance use disorder (Table 3).

**Table 3:** Distribution of patients with Schizophrenia and Bipolar disorder by their substance use disorders (schizophrenia, *n* = 270 and bipolar disorder, *n* = 272) Amanuel Mental Specialized Hospital, Addis Ababa, Ethiopia, August, 2016

Substance use	Schizophrenia		Bipolar disorder	
Disorders	Current use disorder	Life time use (ever had used) disorder	Current use disorder	Life time use (ever had used) disorder
Alcohol use disorder	71 (27.3%)	99 (36.6%)	74 (28.4%)	107 (39.1%)
Khat/chat use disorder	123 (47.3%)	125 (46.3%)	130 (49.8%)	137 (50.3%)
Nicotine use disorder	34 (13.1%)	34 (13.1%)	33 (12.6%)	34 (13%)
Cannabis use disorder	4 (1.5%)	4 (1.5%)	4 (1.5%)	4 (1.5%)
Any substance use disorder	160 (61.5%)	165 (63.5%)	167 (64%)	172 (65.9%)
Poly substance use disorders	95 (36.5%)	130 (48.1%)	71 (27.2%)	86 (31.6%)

## Discussion

This study revealed that the magnitude of suicidal ideation and suicide attempts in patients with schizophrenia and bipolar disorder was comparable with study conducted in high-income country settings **[**13, 14, 22, 24]. In the current study, 119 (44.1%) of schizophrenic participants and 128 (47.1%) of bipolar participants have suicidal ideation and, 56 (20.58%) of schizophrenic participants and 58 (21.32%) of bipolar participants have suicidal attempt. This finding is in agreement with other studies [13, 22, 24].

In this study, both suicidal ideation and attempt were more commonly seen in people with bipolar disorder compared to those with schizophrenia. This finding is in agreement with other studies that reported significantly higher rates of suicide ideation and attempt among patients with bipolar disorder [13, 14, 22, 24].

Suicidal ideation and attempt are common among patients with schizophrenia and bipolar disorder as compared to evidences suicidal ideation and attempt in general population. These findings are in line with other studies that revealed significantly higher suicidal ideation and attempt in patients with severe mental disorder than general population [9, 11, 12, 14].

Our study revealed that patients with severe mental disorders are using different substances. This finding is in line with other studies [20] but higher than [31**]** and lower than [32, 33]. The possible reasons for this difference might be due to the difference in data collection instrument, socio-demographics and culture. Unlike other studies [32–35**]**, 132 (50.6%) (bipolar patients) and 125 (48.1%) (schizophrenic patients), had used khat in their life time. The possible reasons for this difference might be due to differences in socio-demographics and culture.

## Conclusion

Suicidal ideation and attempt were more commonly seen in people with bipolar disorder compared to those with schizophrenia. Co-morbid substance use disorder was a more frequent phenomenon among patients with suicidal ideation and attempt than those without suicidal ideation and attempt was identified in the current study that majority of those who have history of suicidal ideation and attempt have co-occurring substance use disorders as compared to those who have no suicidal ideation and attempt. Co-morbid substance use disorders are common in person with suicidal ideation and attempt. As a result, this indicates the need for further screening and attention of co-morbidity in persons with suicide. Further studies concerning effects and specific relationships between suicide and co-morbid substance use disorders and exploring other factors are recommended.

## Limitation of study

This study only assessed the descriptive part. It will be better to asses factors associated with suicidal ideation and attempt.
